# Effect of the one-day fasting on cortisol and DHEA daily rhythm regarding sex, chronotype, and age among obese adults

**DOI:** 10.3389/fnut.2023.1078508

**Published:** 2023-02-06

**Authors:** Martyna Marciniak, Maki Sato, Rafał Rutkowski, Agnieszka Zawada, Aldona Juchacz, Dagmara Mahadea, Marian Grzymisławski, Agnieszka Dobrowolska, Edyta Kawka, Katarzyna Korybalska, Andrzej Bręborowicz, Janusz Witowski, Dominika Kanikowska

**Affiliations:** ^1^Department of Pathophysiology, Poznan University of Medical Sciences, Poznan, Poland; ^2^Department of Gastroenterology, Dietetics and Internal Diseases, Poznan University of Medical Science, Poznan, Poland; ^3^Institutional Research, Aichi Medical University School of Medicine, Nagakute, Japan; ^4^Greater Poland Center of Pulmonology and Thoracic Surgery of Eugenia and Janusz Zeyland, Poznan, Poland; ^5^Collegium Medicum, Zielona Góra University, Zielona Góra, Poland

**Keywords:** cortisol, DHEA, fasting, obesity, rhythm

## Abstract

**Introduction:**

Physiological and biochemical processes in the human body occur in a specific order and show rhythmic variability. Time dependence characterizes the secretion of cortisol and dehydroepiandrosterone (DHEA). One-day fasting implies alternating fasting days and eating days. The study aimed to determine how 24-h fasting affects the daily rhythm of cortisol and DHEA levels in obese people while taking into account gender and chronotype.

**Methods:**

Forty-nine obese patients (BMI 32.2–67.1 kg/m^2^; 25 women and 24 men) underwent a 3-week hospital-controlled calorie restriction diet to reduce body weight. During hospitalization, patients fasted for 1 day, during which only water could be consumed. Samples of whole mixed unstimulated saliva were collected at 2–3-h intervals over a 64-h period and analyzed for cortisol and DHEA by immunoassays. The individual chronotypes were assessed by the morning and evening questionnaire, according to Horne and Östberg. Three components of daily rhythm were evaluated: amplitude, acrophase, and the so-called MESOR.

**Results:**

Cortisol rhythm showed differences in amplitude (*p* = 0.0127) and acrophase (*p* = 0.0005). The amplitude on the fasting day was 11% higher (*p* = 0.224) than the day after. The acrophase advanced on the day of fasting, 48 min earlier than the day before (*p* = 0.0064), and by 39 min to the day after fasting (*p* = 0.0005). In the rhythm of DHEA, differences were found in the MESOR (*p* = 0.0381). The MESOR on the fasting day increased.

**Discussion:**

Our results obtained during 64 consecutive hours of saliva sampling suggest that one-day fasting may affect three components of cortisol and DHEA daily rhythm. Additionally, no differences were found in the daily rhythm between the morning and evening chronotypes and between females and males. Although aging did not influence daily cortisol rhythm, DHEA amplitude, MESOR, and acrophase changed with age. To the best of our knowledge, this is the first presentation of changes in DHEA rhythm during one-day fasting.

## Introduction

Rhythmicity seen in many processes, including metabolism, reveals both individual habits (e.g., sleep and mealtimes) and the effect of internal body clocks (1). The central pacemaker controls behavioral, metabolic, and physiological rhythms and can synchronize the peripheral oscillators (2). The lack of synchronization between the central clock and peripheral clocks, e.g., by changing the timing of food intake and the diet composition, may lead to the desynchronization of rhythms and the development of metabolic disorders, including obesity or type 2 diabetes (3, 4). Synchronization mechanisms imply various humoral signals, e.g., circulating components such as glucocorticoids (5). Cortisol and DHEA belong to the group of steroid hormones (6, 7). The circadian rhythm of cortisol and DHEA secretion is regulated by the central pacemaker, the so-called biological clock located in the suprachiasmatic nucleus (SCN) of the hypothalamus, and is dependent on the time of sleep and wakefulness (8, 9). Circadian variation in DHEA is also documented, with peaks occurring in the early morning hours (10–12). DHEA is secreted synchronously with cortisol in response to corticotropin-releasing hormone (CRH) and adrenocorticotropic hormone (ACTH) (13). Cortisol plays a major role in maintaining the body’s homeostasis, it is involved in the regulation of carbohydrate, lipid, and protein metabolism. It stimulates gluconeogenesis in the liver and inhibits glucose consumption in peripheral tissues. Cortisol increases lipolysis through its catabolic effect, thus altering adipose tissue (14, 15). It has also been reported that a high and long-term rise in cortisol concentration (as opposed to a short-term increase during a one-day fast) results in increased consumption of high-fat snacks and sweets (16). Overstimulation of the hypothalamic–pituitary–adrenal (HPA) axis may play a role in the pathogenesis of diseases coexisting with obesity (17), e.g., it correlates with excessive food intake and causes emotional eating (18). Moreover, excessive levels of glucocorticoids reduce the activity of the satiety hormone – leptin (19). It is a common belief that visceral adipose tissue has the most significant impact on elevated cortisol levels (20, 21). The amount of visceral fat correlates with the increased HPA axis reactivity, especially in the morning and in response to acute stress (21). DHEA has anti-obesity (22), anti-diabetic (23), and anti-atherosclerotic properties (24). The ratio of cortisol to DHEA seems to be of particular importance, including indicators of metabolic and cardiovascular outcomes in obese patients after bariatric surgery (25). DHEA and dehydroepiandrosterone sulfate (DHEA-S) are defined as large reservoirs that easily converting into more potent androgens in peripheral tissues. A decrease in the levels of these reservoirs can lead to health problems such as obesity and insulin resistance by reducing the inhibitory effect of glucocorticoids (26). This is further exacerbated by increased cortisol levels upon aging (27). On the other hand, since DHEA concentration gradually decreases with age, the implementation of replacement therapies of DHEA might be an effective anti-aging therapy (28, 29).

Food intake is one of the synchronizers of the circadian rhythm (30). One-day fasting is one type of intermittent fasting (IF), comprising a fasting day (no foods and drinks of any energy value, or reduction of food consumption to 25% of usual intake, approximately 500 kcal) and an eating day during which you can consume food without restrictions (31). The participants of the one-day fasting study showed a 3–7% higher body weight loss after 2–3 months of intermittent fasting compared to the control group, and improvement in lipid profile, blood pressure, and insulin sensitivity were also observed (32). Fasting increases the serum cortisol concentration by activating the HPA axis (33). Furthermore, this method of feeding improves metabolism, which in turn prolongs the life of animals, as well as slows down the aging process (34). It is believed that a hypocaloric diet can control metabolism by influencing the biological rhythms of metabolic processes (35). It has also been reported that during fasting, cortisol affects the pathways of metabolic processes in the liver, adipose tissue, and skeletal muscles, and also the regulation of clock gene expression, which in turn influences the biological rhythms of metabolic processes (36). DHEA is a weak androgen and an important precursor to androgens in males and oestrogens in females (37). In addition, DHEA counteracts the effect of cortisol on the immune system and cognitive functions (38–40) improvement of cognitive functions, enhancement.

In our study, we aimed to determine the influence of 1-day fasting on the daily rhythm of cortisol and DHEA in patients with obesity. It is hypothesized that 1-day fasting modifies the daily rhythm of cortisol and DHEA in obese individuals.

## Materials and methods

### Study population and study design

A total of 49 patients with obesity, including 25 women and 24 men, were registered. The mean age (±SD) of participants was 48.6 ± 12.8, and the median (min-max) was 47 (26–70). This study enrolled patients with BMI ≥ 30 kg/m^2^, age > 18 years old, who were referred to initiate a weight loss regimen on doctor’s advice. Exclusion criteria were a history of or current disease (central nervous system, psychiatric, active liver disease, immune, gastrointestinal, endocrine, and hematologic), a history of eating disorders (anorexia, bulimia), alcohol/drug abuse, and ongoing antibiotic therapy and steroid therapy, and shift work, night work. Eligible participants must maintain a regular sleep–wake cycle, residing in bed between 22:00 and 24:00 habitually.

**Figure 1:**
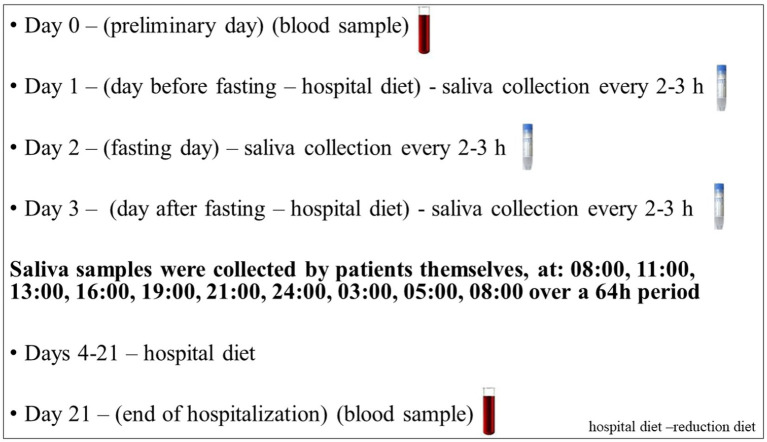
Study protocol. Each subject underwent a protocol schedule with 3-week controlled calorie restriction diet in the hospital. Unstimulated saliva were collected over a 64-h period at 2–3-h intervals starting at 08:00 during the course of three consecutive days for the daily rhythm of cortisol and DHEA. In addition, an initial and final sample of blood on day 0 and day 21 was obtained for biochemical parameters.

Patients were treated for obesity at the Department of Gastroenterology, Dietetics and Internal Diseases of the Poznan University of Medical Sciences. Participants who qualified for the study were subjected to a three-week dietary treatment in a hospital setting. They used an individually selected diet with a 25–30% reduction in the daily caloric supply (reduction of 500–1,000 kcal) in relation to the total energy requirement, calculated according to the Harris and Benedict formula and the physical activity index (41). All patients received the same type of diet provided by a hospital, with the same proportion of nutrients: 20% protein, 25–30% fat, and 50–55% carbohydrates. In addition, on the second day of hospitalization (day 2), patients underwent a 24-h fast with only water intake *ad libitum*. Simultaneously, multiple saliva samplings were collected over a 64-h period at 2–3-h intervals starting at 08:00 (Figure 1).Bodyweight measurement was conducted, including body composition analysis using the BIA electrical bioimpedance method, which allowed the estimation of both the percentage and weight of adipose tissue and muscle tissue in patients (BIA; Tanita/Acern Body Composition Analyzer, Japan).

All patients gave written informed consent and entered the study. Patient baseline characteristics are given in Table 1. The study was approved by the Ethics Committee of the Poznan University of Medical Sciences (No. 249/19) and followed the principles of the Declaration of Helsinki.

**Table 1:** Baseline characteristics of the participants.

Parameters	All participants *n* = 49	Female (F) *n* = 25	Male (M) *n* = 24	*p*-Value F vs. M
Age, years	47 (26–70)	45 (26–68)	49.5 (29–70)	0.3734
Morbid obesity BMI > 40, *n*	29	13	16	0.2952
Glucose intolerance, *n*	20	11	15	0.1931
Diabetes type 2, *n*	6	4	2	0.4087
Dyslipidaemia, *n*	14	7	7	0.9280
Weight, kg	130.2 (89.2–189.4)	112.5 (89.2–189.4)	142.0 (103.3–189.2)	**0.0013**
BMI, kg/m^2^	41.5 (32.2–67.1)	41.6 (32.2–67.1)	41.5 (32.7–57.5)	0.7336
Fat tissue, %	41.6 (32.6–53.2)	43.4 (33.7–53.2)	40.0 (32.6–48.8)	**0.0076**
Fat tissue, kg	51.7 (31.9–100.2)	48.9 (31.9–100.2)	55.2 (34.4–88.7)	0.8729
Visceral fat tissue	18.5 (6–40)	13.5 (6–25)	24.0 (17–40)	**<0.0001**
Muscle tissue, kg	70.3 (31.7–95.6)	59.4 (48.9–84.7)	80.3 (31.7–95.6)	**<0.0001**
Body water (TBW), %	41.3 (33.4–48.6)	43.5 (33.7–53.2)	40.0 (32.6–48.8)	**<0.0001**
Glucose, mg/dl	103.0 (84–313)	103.0 (86–313)	107.5 (84–247)	**0.0473**
Insulin, mU/ml	18.0 (5.8–88.8)	16.9 (5.8–58.3)	23.3 (7.3–88.8)	0.1696
HOMA-IR	5.4 (1.5–49.3)	4.1 (1.5–26.5)	5.8 (1.5–49.3)	**0.0192**
HbA1c, %	6.4 (5–27.4)	5.7 (5–13)	6.1 (5.2–27.4)	0.1504
Cholesterol, mg/dl	180.5 (106–274)	178.5 (112–267)	182.0 (106–274)	0.7984
LDL, mg/dl	106.8 (37.6–204)	103.5 (56.2–142.3)	114.9 (37.6–204)	0.2343
HDL, mg/dl	45.0 (31–100)	49.0 (32–100)	44.0 (31–65)	**0.0146**
Triglycerides, mg/dl	135.0 (50–454)	134.0 (50–454)	137.0 (77–259)	0.5039
CRP, mg/L	6.3 (0.4–16.2)	7.9 (0.4–16.2)	4.0 (0.6–11.5)	**0.0152**
Morning chronotype, *n*	36	20	16	0.5343
Intermediate chronotype, *n*	11	4	7	0.2891
Evening chronotype, *n*	2	1	1	0.9764

The data are given as medians and range (min-max), *n* = 49, day 0. BMI, body mass index; LDL, low-density lipoprotein; HDL, high-density lipoprotein; HOMA-IR, homeostatic model assessment of insulin resistance; CRP, C-reactive protein; HbA1c, glycated hemoglobin. The data were analyzed with the Mann–Whitney test and *X*^2^ test. A value of *p* < 0.05 was considered significant. Bolded values are statistically significant.

### Saliva collection

Unstimulated whole mixed saliva was collected using Salivette swabs (Sarstedt, Nümbrecht, Germany), per the manufacturer’s instructions. All patients were provided with written instructions for the saliva sampling. Samples were collected over a 64-h period at 2-3-h intervals starting at 08:00 (Figure 1). Following centrifugation, the samples of clear saliva were stored at minus 80°C until assayed in batch (9).

### Biochemical measurements

The concentrations of cortisol and DHEA were measured with specific immunoassays from Demeditec Diagnostics GmbH (Kiel, Germany). The sensitivity of the assays was 0.019 ng/ml for cortisol and 6.4 pg/ml for DHEA. Intra- and inter-assay precision for cortisol was (CV%) 7.0 and 7.4%, and for DHEA (CV%) 7.4 and 7.0%, respectively. The assays were performed according to the manufacturer’s instructions. All other measurements were performed by the central laboratory of the university hospital. Insulin resistance was calculated from the fasting levels of blood glucose and insulin using the homeostasis model assessment (HOMA) index (42).

### Questionnaire

An individual’s chronotype was determined using the morningness- eveningness questionnaire (MEQ), as described by Horne and Ostberg (43). This tool could decide on their chronotype, morningness, or eveningness. It may lead to understanding the peak hours of physical and psychological performance and sleep and awakening preferences of these respondents. The subjects were categorized into the morning types, evening types and intermediate types (43).

### Statistical analysis

Statistical analysis was performed using the GraphPad Prism TM 8 software (Software Inc., United States). The normality of the distribution was tested using Shapiro–Wilk’s test. The data that did not follow a Gaussian distribution or ordinal data were analyzed with the Wilcoxon test, Mann–Whitney test or the Kruskal–Wallis test with Dunn’s *post-hoc* test. The relationship between the variables was analyzed with Spearman’s rank correlation coefficient, and categorical data were analyzed with the *X*^2^ test. The results are presented as individual data with the medians and interquartile ranges (min-max). A value of *p* < 0.05 was considered significant. In order to analyze changes over time, for related samples, the ANOVA, two-way ANOVA and appropriate *post-hoc* tests were used. The cortisol and DHEA results were assessed in terms of outliers’ values in the Grubbs’ test. Therefore, in the entire further study, the cortisol and DHEA results were presented without outliers.

### The cosine analysis

The daily rhythm was assessed by a single cosine test using MemCalc/Win (GMS, Tokyo, Japan). The daily rhythm is described by the midline estimating statistic of rhythm (MESOR), amplitude, and acrophase. The MESOR is the mean of all values across the circadian rhythm. The amplitude is half the difference between the highest and the lowest points of the cosine function, best fitting the data. The acrophase represents the time point when the circadian cycle reaches the peak value.

## Results

### Description and distribution of the obese subjects

Almost 60% (59%) of participants (*n* = 29) enrolled in the study had morbid obesity with BMI above 40 kg/m^2^. Among them, 40.8% (*n* = 20) had glucose intolerance, 12% (*n* = 6) diabetes type 2, and 29% (*n* = 14) dyslipidemia. The females were predominant (51%) among the study group. As expected, female patients were found to have a lower 26% body weight, 35% muscle tissue, and 3.5% body water with 3.4% higher fat tissue % as compared with male participants. Female patients had lower glucose, insulin resistance (HOMA) index, and higher HDL and CRP concentrations than men (Table 1).

### Patients’ chronotypes

Patients could be classified as morning chronotypes 73%, (*n* = 36), evening chronotypes 5%, (*n* = 2) and 22%, (*n* = 11) and intermediate chronotypes (Table 1). Due to the small number of participants with the pure evening chronotype, for this analysis, the patients with intermediate and evening chronotypes were grouped together and designated further as evening chronotypes. These patients were compared to those with the morning chronotype. Such an approach was used previously in other studies (34, 35).

### Saliva samples

#### Cortisol

**Figure 2:**
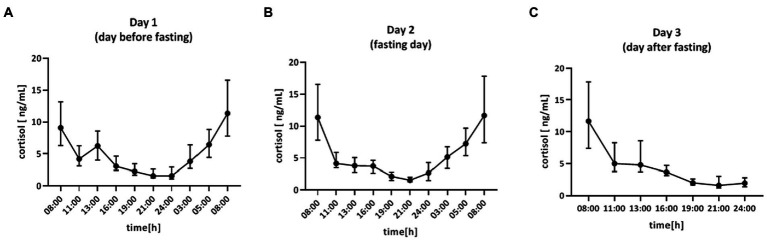
Time course of cortisol concentration (ng/ml) for 64 h in: **(A)** day 1 (day before fasting), **(B)** day 2 (fasting day), **(C)** day 3 (day after fasting), *n* = 49. Each value is the median (min-max).

Saliva samples were collected during the 64-h study (Figure 2). The cosinor method was used to assess the biological rhythm of cortisol. Salivary concentrations of cortisol measured over a 24-h period displayed significant fluctuations. The cosine analysis revealed that these changes exhibited a daily rhythm, and the characteristics of these rhythms are given in Table 2. Differences in amplitude (*p* = 0.0127) and acrophase (*p* = 0.0005) were noticed. The amplitude on day 2 (fasting day) was 11% (*p* = 0.224) higher than the amplitude on day 3 (the day after fasting). On the other hand, acrophase was shifted on the fasting day, 48 min earlier compared to day 1 (*p* = 0.0064) and by 39 min from day 3 (*p* = 0.0005; Table 2). After dividing the patients by gender, differences in amplitude (*p* = 0.03) and acrophase (*p* = 0.0006) were observed in the group of women. The amplitude on the fasting day was 12% higher compared to day 1 and 13% higher on day 3 (the day after fasting). Furthermore, the acrophase was shifted on the fasting day by 66 min compared to day 1 (*p* = 0.0008) and by 48 min to day 3 (*p* = 0.0374). However, no differences were found in the cortisol rhythm between men and women (Table 3). The study group was divided according to chronotype. Differences were noticed for acrophase (*p* = 0.025) in people with morning chronotype. Acrophase was shifted on the fasting day by 57 min earlier than day 1 (*p* = 0.0202) and by 42 min compared to day 3 (*p* = 0.0034). No differences were found in the cortisol rhythm between the morning and evening chronotypes (Table 4). The study group was also divided according to age. No differences were found in the daily rhythm of cortisol between younger (<50 years old) and older (>50 years old) participants (Table 5).

**Table 2:** The characteristics of daily cortisol and DHEA rhythm during 64 h.

Parameters	Day 1 (D1)	Day 2 (D2)	Day 3 (D3)	*p*	D1 vs. D2p	D1 vs. D3p	D2 vs. D3p
Cortisol *n* = 49							
MESOR, ng/ml	4.973 (2.632–7.83)	5.025 (2.543–7.05)	5.175 (1.208–10.84)	0.3288	ns	ns	ns
Amplitude, ng/ml	3.724 (0.1374–10.23)	4.289 (0.1839–10.96)	3.801 (0.275–8.808)	**0.0127**	ns	ns	**0.0224**
Acrophase, clock time	08:24 (04:42–10:48)	07:36 (05:00–10:24)	08:15 (06:18–11:54)	**0.0005**	**0.0064**	ns	**0.0005**
DHEA, *n* = 15							
MESOR, pg/ml	55.25 (11.4–393.4)	60.52 (7.34–435.4)	45.0 (10.35–277.2)	**0.0381**	ns	ns	ns
Amplitude, pg/ml	32.12 (2.963–235.6)	42.85 (4.924–176.5)	30.2 (1.75–128.9)	0.2818	ns	ns	ns
Acrophase, clock time	5:14 (1:58–20:44)	5:13 (00:04–10:05)	7:00 (2:08–20:26)	0.0569	ns	ns	ns
Cortisol/DHEA ratio, *n* = 15							
05:00	56.5 (7.7–949.6)	194.4 (9.7–1891)	–	–	**0.043**	–	–
08:00	87 (7.7–373)	101.3 (11.2–2,284)	255.4 (5.7–3,038)	0.3441	ns	ns	ns
24:00	48.3 (4.8–1842)	24.5 (3.8–2,939)	30.5 (5.2–6,122)	0.7659	ns	ns	ns

The data are given as medians and ranges (min-max). The daily rhythm is described by the midline estimating statistic of rhythm (MESOR), amplitude, and acrophase. The MESOR is the mean of all values across the circadian rhythm. The amplitude is half the difference between the highest and the lowest points of the cosine function, best fitting the data. The acrophase represents the time point when the circadian cycle reaches the peak value. DHEA, dehydroepiandrosterone; ns, non-significant. The data were analyzed with a one-way ANOVA test. A value of *p* < 0.05 was considered significant. Bolded values are statistically significant.

**Table 3:** The characteristics of daily cortisol and DHEA rhythm during 64 h according to sex.

Parameters	Female (F)							Male (M)							F vs. Mp
	Day 1 (D1)	Day 2 (D2)	Day 3 (D3)	*p*	D1 vs. D2p	D1vs. D3p	D2 vs. D3p	Day 1 (D1)	Day 2 (D2)	Day 3 (D3)	*p*	D1 vs. D2p	D1 vs. D3p	D2 vs. D3p	
Cortisol															
	*n* = 25							*n* = 24							
MESOR, ng/ml	4.816 (2.632–7.83)	4.842 (2.543–7.04)	4.885 (2.841–7.2)	0.5806	ns	ns	ns	5.185 (3.201–7.38)	5.08 (2.719–7.04)	5.453 (2.557–10.48)	0.4059	ns	ns	ns	ns
Amplitude, ng/ml	3.812 (0.137–8.747)	4.511 (0.1839–10.96)	3.6 (0.275–9.808)	**0.03**	ns	ns	ns	3.585 (1.543–6.614)	3.876 (0.6909–7.004)	4.298 (0.6294–8.407)	0.0912	ns	ns	ns	ns
Acrophase, clock time	08:18 (04:42–10:48)	07:12 (05:18–09:24)	08:00 (07:06–09:48)	**0.0006**	**0.0008**	ns	**0.0374**	08:33 (05:36–10:36)	07:54 (05:36–10:18)	08:30 (06:18–11:54)	0.0865	ns	ns	ns	ns
DHEA															
	*n* = 8							*n* = 7							
MESOR, pg/ml	51.46 (14.18–323.9)	67.31 (20.63–269.8)	49.41 (11.7–211.9)	0.1495	ns	ns	ns	55.25 (11.4)	51.29 (7.34–435.4)	42.32 (10.35–277.2)	0.1916	ns	ns	ns	ns
Amplitude, pg/ml	27.92 (2.963–122.5)	35.62 (6.135–130.6)	30.99 (3.271–84.82)	0.7943	ns	ns	ns	44.79 (6.105–235.6)	44.57 (4.924–176.5)	30.2 (1.75–128.9)	0.4861	ns	ns	ns	ns
Acrophase, clock time	6:06 (02:35–20:44)	5:06 (00:35–8:30)	8:10 (3:22–20:26)	0.0789	ns	ns	ns	5:14 (1:58–13:22-)	6:56 (00:04–10:05)	4:51 (02:08–11:08)	0.6197	ns	ns	ns	ns

The data are given as medians and ranges (min-max). DHEA, dehydroepiandrosterone; ns, non-significant. The data were analyzed with a one-way ANOVA test and with two-way ANOVA for female vs. men. A value of *p* < 0.05 was considered significant. Bolded values are statistically significant.

**Table 4:** The characteristics of daily cortisol and DHEA rhythm during 64 h according to chronotype.

Parametrs	Morning chronotype (M)							Evening chronotype (E)							M vs. Ep
	Day 1 (D1)	Day 2 (D2)	Day 3 (D3)	*p*	D1 vs. D2p	D1 vs. D3p	D2 vs. D3p	Day 1 (D1)	Day 2 (D2)	Day 3 (D3)	*p*	D1 vs. D2p	D1 vs. D3p	D2 vs. D3p	
Cortisol															
	*n* = 36							*n* = 13							
MESOR, ng/ml	4.932 (2.632–7.83)	4.738 (2.543–7.04)	5.061 (2.557–7.1)	0.931	ns	ns	ns	5.18 (3.94–7.25)	5.55 (4.134–6.42)	6.054 (3.603–7.7)	0.783	ns	ns	ns	ns
Amplitude, ng/ml	3.243 (0.1374–9.06)	4.083 (0.1839–10.96)	3.579 (0.275–8.407)	0.065	ns	ns	ns	4.756 (1.961–7.187)	4.489 (1.439–9.794)	4.344 (0.6796–8.808)	0.2108	ns	ns	ns	ns
Acrophase, clock time	08:33 (04:42–10:48)	07:36 (05:00–10:24)	08:18 (06:18–11:54)	**0.025**	**0.0202**	ns	**0.0034**	08:18 (05:36–10:12)	07:42 (05:30–08:42)	08:06 (07:06–10:12)	0.1803	ns	ns	ns	ns
DHEA															
	*n* = 10							*n* = 5							
MESOR, pg/ml	72,15 (14.18–393.4)	67.31 (19.51–435.5)	49.41 (26.84–277.7)	0.3675	ns	ns	ns	31.81 (11.4–121.0)	38.07 (7.34–176.8)	15.52 (10.35–107.3)	0.0934	ns	ns	ns	ns
Amplitude, pg/ml	47.97 (2.963–235-6)	43.71 (14.82–176.5)	38.78 (20.42–128.9)	0.8302	ns	ns	ns	23.59 (6.105–129.5)	10.1 (4.924–123.3)	11.57 (1.75–84.82)	0.0394	ns	0.0342	ns	ns
Acrophase, clock time	4:50 (1:47–20:44)	3:44 (00:04–8:30)	8:10 (2:08–15:41)	0.0456	ns	ns	0.0417	6:48 (5:08–13:22)	6:11 (4:48–10:05)	6:44 (4:51–20:26)	0.9537	ns	ns	ns	ns

The data are given as medians and ranges (min-max). DHEA, dehydroepiandrosterone; ns, non-significant. The data were analyzed with a one-way ANOVA test and with two-way ANOVA for morning chronotype vs. evening chronotype. A value of *p* < 0.05 was considered significant. Bolded values are statistically significant.

**Table 5:** The characteristics of daily cortisol and DHEA rhythm during 64 h according to age.

Parameters	Age < 50 years							Age > 50 years							
	Day 1 (D1)	Day 2 (D2)	Day 3 (D3)	*p*	D1 vs. D2p	D1 vs. D3p	D2 vs. D3p	Day 1 (D1)	Day 2 (D2)	Day 3 (D3)	*p*	D1 vs. D2p	D1 vs. D3p	D2 vs. D3p	<50 years vs. >50 yp
Cortisol															
	*n* = 27							*n* = 22							
MESOR, ng/ml	5 (0.7–9.62)	5.1 (0.8–9.9)	5.22 (1.21–12.85)	ns	ns	ns	ns	5.69 (2.63–12.55)	5.3 (2.54–12.29)	5.15 (2.36–10.84)	ns	ns	ns	ns	ns
Amplitude, ng/ml	3.81 (0.14–10.23)	4.36 (0.18–10.96)	3.42 (0.28–10.17)	ns	ns	ns	ns	3.66 (1.04–9.06)	4.07 (1.05–9.8)	4.15 (0.63–8)	ns	ns	ns	ns	ns
Acrophase, clock time	8:17 (4:04–22:17)	7:35 (5:17–22:11)	8:35 (7:04–13:04)	**0.0391**	ns	ns	ns	8:17 (2:35–10:46)	7:56 (3:22–9:22)	8:11 (6:17–11:49)	ns	ns	ns	ns	ns
DHEA															
	*n* = 7							*n* = 8							
MESOR, pg/ml	121 (32.51–3,939)	141.8 (51.29–435.4)	101.8 (42.32–277.2)	ns	ns	ns	ns	30.88 (11.4–111.9)	34.36 (7.34–112)	26.34 (10.35–45.76)	ns	ns	ns	ns	<0.01
Amplitude, pg/ml	122.5 (23.71–235.6)	48.02 (14.82–176.5)	78.88 (29.44–128.9)	ns	ns	ns	ns	17.2 (2.96–88.39)	19.27 (4.92–48.73)	30.88 (11.4–111.9)	ns	ns	ns	ns	<0.01
Acrophase, clock time	5:11 (2:35–8:22)	4:56 (2:04–8:32)	8:35 (3:11–1:04)	ns	ns	ns	ns	6:17 (1:56–20:39)	5:39 (00:04–10:06)	5:49 (2:04–20:23)	ns	ns	ns	ns	<0.01

The data are given as medians and ranges (min-max). DHEA, dehydroepiandrosterone; ns, non-significant. The data were analyzed with a one-way ANOVA test and with two-way ANOVA for age < 50 years vs. age > 50 years. A value of *p* < 0.05 was considered significant. Bolded values are statistically significant.

**Figure 3:**
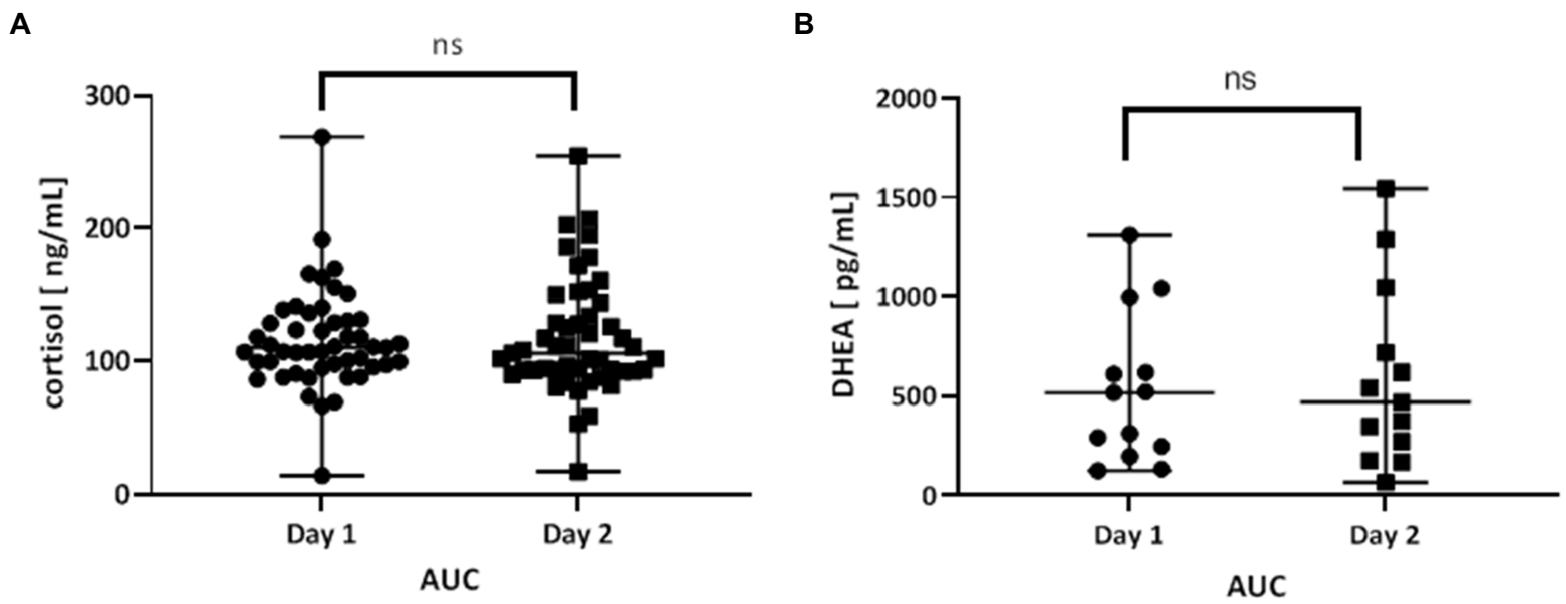
**(A)** Area under the curve (AUC) of the time course of cortisol levels day 1 (day before fasting) and day 2 (fasting day). The AUC was calculated for each patient separately (*n* = 49), and the mean value of day 1 and day 2 were compared. **(B)** Area under the curve (AUC) of the time course of DHEA levels day 1 (day before fasting) and day 2 (fasting day). The AUC was calculated for each patient separately (*n* = 15), and the mean value of day 1 and day 2 were compared. Values are expressed as median (min-max). A value of *p* < 0.05 was considered significant. The data were analyzed with the Wilcoxon test.

The area under the curve (AUC) for cortisol concentration was calculated to compare the total concentration of cortisol on day 1 and day 2. There were no differences in AUC (*p* = 0.8766; Figure 3A).

#### DHEA

**Figure 4:**
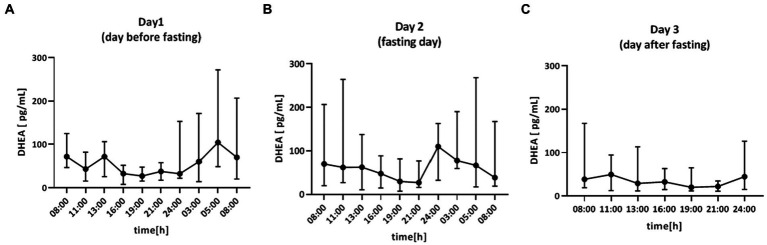
Time course of DHEA concentration (pg/ml) for 64 h in: **(A)** day 1 (day before fasting), **(B)** day 2 (fasting day), **(C)** day 3 (day after fasting), *n* = 15. Each value is the median (min-max).

Saliva samples for DHEA were collected during the 64-h study (Figure 4). The cosine analysis revealed that these changes exhibited a daily rhythm, and the characteristics of these rhythms are given in Table 2. Differences in MESOR (*p* = 0.0381) were noticed. The MESOR on day 2 (fasting day) was 11% higher compared to the MESOR on day 1 (the day before fasting) and 13.4% higher than the MESOR on day 3 (the day after fasting). No differences were found in the DHEA rhythm in the group of women, men and between these two groups (Table 3). After dividing the patients by chronotype differences in acrophase, were observed (*p* = 0.456) in the morning chronotype group and amplitude (*p* = 0.0394) evening chronotype group (Table 4). The acrophase was shifted on the fasting day by 66 min compared to day 1 and by 266 min to day 3 (*p* = 0.0417). The amplitude on day 2 (fasting day) was 57.2% lower than the amplitude on day 1 and 12.7% lower than the amplitude on day 3. No differences were found in the DHEA rhythm between the morning and evening chronotypes (Table 4). The study group was divided according to age. The differences were found in the parameters of DHEA rhythm between younger (<50 years old) and older (>50 years old) participants (*p* < 0.01, 2-way ANOVA). The MESOR and amplitude in older patients were reduced, and the acrophase was delayed (Table 5).

The area under the curve (AUC) for DHEA concentration was calculated to compare the total concentration of DHEA on day 1 and day 2. There were no differences in AUC (*p* = 0.6355; Figure 3B).

### Cortisol/DHEA ratio

The cortisol to DHEA ratio for all time points of saliva sampling was calculated.

Only in the morning at 05:00 did the ratio differ between pre-fasting and fasting day, whereas no significant differences could be found between any other time points of the experimental days (Table 2).

### Calorie restriction

The 3-week calorie restriction resulted in a modest but significant reduction in body weight and – consistently – in BMI, %, kg of adipose tissue, visceral fat, body water, and muscle tissue (Table 6). In addition, a more significant decrease in glycated hemoglobin and total cholesterol, and triglyceride levels was observed (Table 6). Spearman correlations between cortisol and DHEA rhythm and changes in anthropological and biochemical parameters after dietary restriction were calculated. There were no correlations between those parameters.

**Table 6:** Anthropometric and metabolic characteristics of the study group (*n* = 49), taking into account measurement changes before (day 0) and after caloric restriction (day 21).

Parameters	The obese before caloric restriction *n* = 49	The obese after caloric restriction *n* = 49	*p*-Value
Weight, kg	130.2 (89.2–189.4)	125.8 (84.6–184.2)	**<0.0001**
BMI, kg/m^2^	41.5 (32.2–67.1)	40.6 (30.0–62.7)	**<0.0001**
Fat tissue, %	41.6 (32.6–53.2)	40.5 (29.8–55.1)	**0.0001**
Fat tissue, kg	51.7 (31.9–100.2)	48.8 (29.2–97.6)	**<0.0001**
Visceral fat tissue	18.5 (6.0–40.0)	18.0 (5.0–36.0)	**<0.0001**
Muscle tissue, kg	70.3 (31.7–95.6)	70.0 (47.9–4.1)	**0.001**
Body water (TBW), %	41.3 (33.4–48.6)	40.5 (29.9–55.1)	**<0.0001**
Glucose, mg/dl	103.0 (84.0–313.0)	102.0 (84.0–363.0)	0.6461
Insulin, mU/ml	18.0 (5.8–88.8)	22.1 (6.2–41.7)	0.0799
HOMA-IR	5.4 (1.5–49.3)	5.5 (1.6–10.9)	0.0934
HbA1c, %	6.4 (5.0–27.4)	5.7 (5.0–5.8)	**0.0053**
Cholesterol, mg/dl	180.5 (106.0–274.0)	166.5 (87.0–266.0)	**0.0244**
LDL, mg/dl	106.8 (37.6–204.0)	47.0 (28.0–82.0)	0.144
HDL, mg/dl	45.0 (31.0–100.0)	97.9 (39.0–198.0)	0.2576
Triglycerides, mg/dl	135.0 (50.0–454.0)	118.0 (38.0–438.0)	**0.0237**
CRP, mg/L	6.3 (0.4–16.2)	4.6 (0.3–28.9)	0.4614

BMI, body mass index; LDL, low-density lipoprotein; HDL, high-density lipoprotein; HOMA-IR, homeostatic model assessment of insulin resistance; CRP, C-reactive protein; HbA1c, glycated haemoglobin. The data were analyzed with the Mann–Whitney test. A value of *p* < 0.05 was considered significant. Bolded values are statistically significant.

## Discussion

The present study assessed the daily rhythm of cortisol and DHEA in obese subjects following a one-day fasting diet. There are only very few scientific reports on the daily rhythm of DHEA. To the best of our knowledge, this is the first study to assess the relationship between fasting and DHEA rhythm. On the fasting day, we found differences in the amplitude and acrophase of the daily cortisol rhythm and the MESOR of the daily DHEA rhythm. We have shown that one-day fasting causes significant changes between the minimum and maximum values of the cortisol rhythm curve. The amplitude on the fasting day was higher than on the other sampling days. Moreover, a shift in the phase of the cortisol rhythm has been observed. Acrophase on the fasting day occurred earlier. The MESOR of the cortisol rhythm did not differ between days.

In a partially contradictory study by Bergendahl et al. (44), the authors also noticed that fasting caused an increase in amplitude and MESOR of the circadian rhythm of serum cortisol; however, it did not affect the acrophase. The duration of fasting was identical to our study and amounted to 24 h, though the study group consisted of slim men. In another study involving lean people and overweight patients, an 84-h fast was implemented, and an increase of the MESOR and the amplitude of the cortisol rhythm was observed (44). A plausible explanation for the discrepancies in the results of MESOR and acrophase with our study could be due to the differences in the study protocol, i.e., duration of fasting (5 days vs. 1 day fast) and different characteristics of the subjects (lean vs. obese). However, in all studies, the amplitude of the cortisol rhythm was increased. The suprachiasmatic nucleus of the hypothalamus drives the 24-h pattern in cortisol (45). The animal study by Girotti et al. (46) showed that the feeding cue is a strong synchronizer of the HPA axis. In their review, Nakamura et al. (33) emphasize that fasting has a powerful effect on increasing cortisol secretion. In this respect, our results obtained during several consecutive days of saliva sampling might confirm that fasting may elicit changes in the cortisol rhythm.

Obesity is associated with increased inactivation of cortisol and impaired hepatic regeneration of cortisol from cortisone (47). Moreover, obesity is accompanied by an altered circadian rhythm of cortisol (48, 49). On the other hand, weight loss associated with a very low-calorie diet (VLCD) in obese people normalizes the production of cortisol (48, 50, 51). In the study by van Rossum et al. (52), a moderate calorie restriction diet for 6 months did not affect the circadian cortisol rhythm.

According to Al Safi et al. (48), the cortisol concentration in obese patients was significantly higher compared to lean individuals. Furthermore, obese subjects showed no decrease in cortisol concentration in the evening. In contrast, the study by Parra et al. (51) showed that in obese men, the secretion of cortisol in the fasting state and after a meal was lower than in lean subjects. Our study showed that the total cortisol concentration measured by AUC did not change during fasting (Figure 3). The current research indicates a relationship between fasting and the circadian cortisol rhythm. The observed alteration in cortisol rhythm might indicate disturbances in regulating the HPA axis under the influence of fasting.

When analyzing the study group according to gender, we found that female participants presented alterations in the amplitude and acrophase of the cortisol rhythm. The amplitude of the rhythm on the fasting day was higher, and the acrophase occurred earlier. There were no differences in the MESOR parameter. In contrast, none of the parameters characterizing the cortisol rhythm differed in the male subjects.

A study by Vance et al. (53) examined the effect of 5-day fasting on cortisol secretion in lean men. The authors noted changes in the amount of cortisol, and the amplitude of the circadian rhythm decreased (53). Multiple studies have been inconclusive, with some reports showing a higher cortisol concentration in women and others in men.

One of the hypotheses explaining these differences may be the use of different biological materials in the studies. In the research that assessed sera or blood plasma, the cortisol concentration was higher in men (54, 55), while an inverse relationship was observed in studies involving saliva.

Similar findings have been confirmed in the survey by Kudielka et al. (56), where the authors demonstrated that the total plasma cortisol concentration was higher in older women than in older men, while the opposite was observed for salivary cortisol levels.

A possible justification for the differences the cortisol concentration between genders could be that, i.e., exogenous estrogens taken by women can increase the concentration of corticosteroid-binding globulin in the blood (57, 58). Additional reports suggest that birth control pills may cause increased free cortisol through increased basal activation of the HPA axis (59, 60).

Other theories suggest a difference in the occurrence of chronotypes depending on gender. Some studies have shown that women are more of a “morning chronotype” than men, have an earlier phase of the biological clock gene expression rhythm, and have a shorter period of the circadian rhythm (61–63). Moreover, women have an earlier occurrence of acrophase in the circadian cortisol rhythm compared to the cortisol rhythm in men (58). The current study found only differences in acrophase in the morning chronotype participants. On the fasting day, acrophase occurred earlier; no differences were observed for the other rhythm parameters. Similarly, the rhythm was no different in the evening chronotypes. Finally, no differences were reported in the rhythms between the morning and evening chronotypes. Also, Toda et al. (64) found no differences in the circadian rhythm of cortisol concentration between the morning and evening chronotypes. On the other hand, Kudielka et al. (65) have reported that individuals with a morning chronotype may have higher cortisol levels upon awakening. These inconsistencies may be related to the differences in survey methodology. When we compared MESOR, amplitude, and acrophase of cortisol daily rhythm in the group of younger (below 50 years old) and older (above 50 years old) participants no differences were found. The results are similar to those reported in published literature (29).

The current study also assessed the rhythm of DHEA secretion. We noted that the MESOR of DHEA rhythm on fasting day increased. Furthermore, after dividing the patients by chronotype, there were differences in acrophase in the morning chronotypes and amplitude in the evening chronotypes. On fasting day, the acrophase was shifted earlier in the morning chronotypes, while in evening chronotypes, the amplitude was lower.

Lastly, we found no differences in the pattern of DHEA rhythm between the morning and evening chronotypes and between men and women. There are few reports that DHEA concentrations are usually higher in women than men (13), but others did not find differences in gender (10). When we compared MESOR, amplitude, and acrophase of DHEA rhythm in the group of women and men we could not find any differences.

However, we noted that all measured parameters of DHEA rhythm differed in younger and older patients with a trend toward a decrease in MESOR, amplitude, and delayed acrophase with age. Touitou (66), in his review described a similar reduction in DHEA rhythm, related to alterations in the biosynthesis pathways of adrenal steroids.

To date, there are only a few studies on DHEA changes in a fasting diet. A study by Harvie et al. (67) involving obese women on an IF diet for 6 months and consuming 25% energy demand for 2 days noted no difference between the concentration of DHEA before and after the dietary intervention.

In a different study, Jakubowicz et al. (68) compared the effects of consuming less than 50% of calories at dinner compared to eating more than 50% of calories at breakfast in women with polycystic ovary syndrome (PCOS) and found that DHEA was reduced by 35% in the breakfast group compared to the dinner group. Jakubowicz et al. (69) investigated the effects of a very low (1,000 kcal) and moderate (1,400 kcal) calorie restriction diet for 2 months on serum DHEA-S concentration in obese patients. The diet resulted in an increase in DHEA –S in obese men but not women.

Cortisol and DHEA are steroid hormones with opposing effects. In obese patients, there is an increased concentration of cortisol, which in turn can cause a decrease in DHEA concentrations (25). We evaluated the cortisol/DHEA ratio at multiple time points of saliva sampling. The ratio was similar on all days, with only a different concentration in the early morning (05:00) of the fasting day. The ratio was higher, due to their lower DHEA concentration and higher cortisol level. Moreover, patients were treated for 3 weeks in a hospital setting with a moderate calorie restriction diet, which allowed reduced anthropometric parameters such as body mass, BMI, and fat mass but also muscle mass. Reduced body mass resulted in improving, HbA1c, and lipids concentration (cholesterol, triglycerides). Correlations between cortisol and DHEA rhythm and changes in anthropological and biochemical parameters after dietary restriction were calculated. No correlations were found between those parameters.

The impact that a calorie restriction diet may have on the relationship between cortisol and DHEA rhythms and changes in metabolic parameters and body weight after the restriction is limited. The published studies have focused on the correlation between obesity and those hormone levels. In the study by Abraham et al. (70) on obese patients, it was observed that the concentration of cortisol in urine samples and after the dexamethasone test did not correlate with patients’ BMI. However, it was reported that cortisol in saliva tended to increase with increasing BMI. Similarly, Travison et al. (71), Jackson and Steptoe (72), and Al-Safi et al. (48) noted a relationship between BMI and cortisol concentration. Furthermore, Reynolds et al. (73) pointed out that cortisol concentration in men was inversely related with BMI. Studies by Travison et al. (71) and Jackson and Steptoe (72) were conducted with a considerable number of patients. Harithy (74) showed a negative relationship between the BMI value and DHEA-S concentration in lean and obese women. In addition, in obese patients, the level of DHEA-S showed a positive correlation with insulin concentration. There was no significant association between DHEA-S and glucose levels (74). Oltmanns et al. (75) and Reynolds et al. (73) noticed a positive correlation between cortisol concentration and glucose concentration (73, 75).

The limitations in our current study are inadequate knowledge of oral contraceptives used by patients, which may have affected cortisol concentration (76). In addition, the DHEA concentration was measured only in 15 patients due to a shortage of our funding.

Moreover, the study was performed with a relatively small sample size, and the analysis according to age range could not be achieved by sex because the number of DHEA measurements was deficient. Further studies in a larger sample of patients will help better ascertain the effect on the ratio of Cortisol/DHEA of IF. Therefore, this study would be the first approach to the impact of IF on DHEA rhythm.

In conclusion, one-day fasting affects the daily rhythms of cortisol and DHEA in obese patients. Nevertheless, very little is known about circadian rhythm-regulated secretion of DHEA and cortisol by a fasting diet. The regulation of the body clock seems to be mediated by fasting, but the mechanism is not yet fully understood. Additional studies are needed to elucidate the regulation of the body clock, daily rhythms of cortisol, and DHEA by fasting diet. Understanding the alteration of daily hormonal rhythms influenced by fasting could lead to developing new treatments for obesity and its comorbidities, which are serious public health concerns.

## Data availability statement

The raw data supporting the conclusions of this article will be made available by the authors, without undue reservation.

## Ethics statement

The studies involving human participants were reviewed and approved by Ethics Committee of the Poznan University of Medical Sciences (No. 249/19). The patients/participants provided their written informed consent to participate in this study.

## Author contributions

MM conceived the study, managed the patients, and collected clinical samples. MS performed statistical analyses. RR and EK performed biochemical analyses. AZ, AJ, MG, and AD managed the participants. DM, KK, AB, and JW critically reviewed the manuscript. DK designed and supervised the study and wrote draft of manuscript. All authors contributed to the article and approved the submitted version.

## Conflict of interest

The authors declare that the research was conducted in the absence of any commercial or financial relationships that could be construed as a potential conflict of interest.

## Publisher’s note

All claims expressed in this article are solely those of the authors and do not necessarily represent those of their affiliated organizations, or those of the publisher, the editors and the reviewers. Any product that may be evaluated in this article, or claim that may be made by its manufacturer, is not guaranteed or endorsed by the publisher.
